# Tatjana Hörnle, Stefan Huster, Ralf Poscher (Hrsg) (2021) Triage in der Pandemie: Mohr Siebeck, Tübingen, 372 Seiten, 34,00 €, ISBN 978-3-16-160201-6

**DOI:** 10.1007/s00481-021-00675-6

**Published:** 2021-11-15

**Authors:** Héctor Wittwer

**Affiliations:** Fakultät für Humanwissenschaften, Lehrstuhl für Praktische Philosophie, Otto-von-Guericke-Universität, Postfach 41 20, 30016 Magdeburg, Deutschland

Seit ihrem Beginn ist die Covid-19-Pandemie von einer intensiven wissenschaftlichen Diskussion begleitet worden. Nicht nur die in der Öffentlichkeit besonders präsente Virologie, sondern auch andere Disziplinen wie die Medizinethik, die Rechtswissenschaft und die Philosophie haben „in Echtzeit“ (Mujerji/Mannino) auf die moralischen, rechtlichen und politischen Herausforderungen reagiert, welche die Pandemie mit sich gebracht hat. Dies belegen zahlreiche Artikel und Themenschwerpunkte in Fachzeitschriften, einige Monographien und etliche Sammelbände. Der hier zu besprechende Band stellt einen weiteren Beitrag zu der noch andauernden Debatte über den richtigen Umgang mit der Pandemie dar. Wie es sein Titel zum Ausdruck bringt, steht dabei das Problem der Triage im Mittelpunkt, d. h. die Frage, ob und gegebenenfalls wie Patienten in Situationen, in denen die Ressourcen nicht dafür ausreichen, alle zu behandeln, priorisiert werden dürfen oder sogar sollen. Wenn man von der plausiblen Annahme ausgeht, dass die Covid-19-Pandemie kein Einzelfall bleiben wird, sondern dass ähnliche Epidemien auch in Zukunft auftreten können, dann wird deutlich, dass die Relevanz dieser Frage weit über die Pandemie, die wir derzeit erleben, hinausreicht.

Dem Problem der Priorisierung in Krisensituationen kann man sich auf verschiedene Weise annähern. In dem vorliegenden Band stehen *die rechtliche und die ethische Perspektive* auf das Problem im Vordergrund. Dies wird an der Zusammensetzung der Autorenschaft deutlich: Neun Beiträge stammen von Juristen und zwei von Ethikern. Tatjana Hörnle und ihren beiden Mitherausgebern ist es gelungen, renommierte Kolleginnen und Kollegen als Beitragende zu gewinnen. Dementsprechend hochkarätig ist das Niveau der Texte.

Im Rahmen dieser Besprechung ist es unmöglich, alle Beiträge im Einzelnen zu referieren. Stattdessen sollen hier die Probleme, die im Mittelpunkt der Diskussion stehen, skizziert und die Fragen genannt werden, an denen sich die Geister scheiden. Die in dem Band behandelten *Themen* lassen sich folgendermaßen systematisieren. Ein *erster* Themenkomplex betrifft die Frage, wie das gegenwärtig in Deutschland geltende Recht in Bezug auf die Triage zu interpretieren ist: Enthält unser Rechtssystem formale oder materiale Vorschriften zur Priorisierung in Krisensituationen? Oder verbietet das Grundgesetz die Triage? Wie ist die Triage strafrechtlich zu bewerten? *Zweitens* wird die Frage behandelt, ob der deutsche Gesetzgeber die Triage rechtlich regeln darf oder ob ihm dies durch das Grundgesetz untersagt ist. Lässt sich verfassungsrechtlich möglicherweise sogar eine Pflicht des Gesetzgebers zur Regelung der Triage begründen? *Drittens* wird von einem rechtsethischen Standpunkt aus die Frage gestellt, welchen Inhalt zukünftige rechtliche Vorschriften zur Triage haben sollten. Neben diesen drei von den Herausgebern ausdrücklich genannten Problemen lässt sich *viertens* eine Frage ausmachen, die im Inhaltsverzeichnis zwar nicht explizit erwähnt wird, die sich aber vorder- oder hintergründig durch den ganzen Band zieht: Wer sollte für die Festlegung der Kriterien für die Priorisierung in Krisensituationen zuständig sein: das Recht oder die Medizin? Hier geht es um die normative Deutungshoheit über den Umgang mit unzureichenden medizinischen Ressourcen in Krisensituationen. In diesem Zusammenhang stellt sich beispielsweise die Frage, ob die von verschiedenen Fachgesellschaften erlassenen klinisch-ethischen Empfehlungen, wie etwa diejenigen der Deutschen Interdisziplinären Vereinigung für Intensiv- und Notfallmedizin (DIVI), mit dem geltenden Recht vereinbar sind. Offensichtlich hängt diese Frage untrennbar mit dem zuerst genannten Problem zusammen: Wenn das Recht Vorschriften enthält, die für die Triage gelten, dann kommt diesen selbstverständlich der Vorrang vor den Empfehlungen der Fachgesellschaften zu. Falls die Triage hingegen in einem *rechtsfreien* Raum angesiedelt sein sollte, stünde es den Fachgesellschaften zumindest* prima facie* frei, diese normative Lücke mit ihren eigenen Empfehlungen zu füllen.

Die Antworten auf diese vier Fragen können hier nicht vollständig wiedergegeben werden. Einige resümierende Bemerkungen müssen genügen. Uneinigkeit besteht bereits in Bezug auf die erste Frage, ob das deutsche Recht Regelungen enthält, die für die Triage relevant sind. Steffen Augsberg vertritt z. B. die These, dass der Schutz der Menschenwürde das Prinzip der „Lebenswertindifferenz“ enthält, dem zufolge der Wert und die vermutliche Dauer eines Lebens keine Rolle bei der Priorisierung spielen dürften. Insofern könne man dem Grundgesetz zumindest negative, formale Vorschriften für die Priorisierung entnehmen. Stefan Huster argumentiert hingegen dafür, dass die Konstellation der Triage zu speziell ist, als dass sie grundgesetzlich geregelt werden könnte.

Was den Inhalt der schon geltenden oder noch zu erlassenden Gesetze betrifft, so kann man eine Mehrheits- und eine Minderheitsposition ausmachen. Die Mehrheitsposition besagt, dass bei der Triage das Lebensalter, die mutmaßliche Lebenserwartung und der Gesundheitszustand der Betroffenen nicht berücksichtigt werden dürfen. Gemäß der Minderheitsposition, die durch Elisa Hoven repräsentiert wird, sind hingegen die „relative Lebenserwartung sowie das Lebensalter eines Patienten legitime Kriterien für die Entscheidung eines Arztes in einer Triage-Situation“ (S. 369). Andere Autoren (Armin Engländer und Till Zimmermann) plädieren dafür, dass die Auswahl der zuerst zu behandelnden Patienten durch ein Zufallsverfahren erfolgen solle.

Die offensivsten Aussagen in dem Band betreffen die Frage, wer für die Regelung der Priorisierung in Krisensituationen zuständig ist: das Recht (der Gesetzgeber) oder die Ärzteschaft. Die klarsten Thesen dazu finden sich bei den Juristen Bijan Fateh-Moghadam und Thomas Gutmann. Ihnen zufolge „stehen die in existentieller Weise grundrechtsrelevanten Entscheidungen über die Priorisierung von Covid-19-Patient/innen […] ebenso wenig wie die Kriterien der Zuteilung von Organen zur Transplantation zur freien Disposition professioneller oder ‚ethischer‘ Selbstregulierung der Medizin“ (S. 294).

Wie dieser knappe Überblick hoffentlich verdeutlicht, dokumentiert der hier zu besprechende Sammelband die aktuellen Debatten über die moralische und rechtliche Zulässigkeit der Triage. Dabei wird deutlich, wo die theoretischen Fronten verlaufen und welche möglichen Antworten es auf die Fragen gibt, die durch die Unumgänglichkeit der Priorisierung in Krisensituationen aufgeworfen werden. Für Nichtjuristen wie den Rezensenten ist die Lektüre des Bandes sicherlich anspruchsvoll. Dennoch kann das Buch den Medizinethiker*innen, die sich mit den Problemen der Triage beschäftigen, die auf der Grenze zwischen Medizinethik und Recht angesiedelt sind, durchaus empfohlen werden.

